# Supplementary material for: Independent Evolution Has Led to Distinct Genomic Signatures in Dutch Elm Disease-Causing Fungi and Other Vascular Wilts-Causing Fungal Pathogens
Source: J Fungi (Basel). 2022 Dec 20;9(1):2. doi: 10.3390/jof9010002 (PMC9864908; doi:10.3390/jof9010002)
Supplement: Supplementary file 1 [file jof-09-00002-s001.zip › SupplementaryFigures.pdf]

Supplementary Figures

Wilt group

Class	Node	Fusarium wilts	Ceratocystis wilts	Bretziella fagacearum	Verticillium wilts	Ophiostoma wilts	Raffaelea/ Leptographium	Raffaelea lauricola
SMC	ANC wilt	474.7	75.7	80	363.7	167.3	280.3	359
SMC	ANC non-wilt	467.8	93.6	89.9	382.9	167.8	289.2	**289.2**
SMC	non-wilt	487.3	99	66	**257**	169	353	353
pks	ANC wilt	116.7	21.8	45	118.5	102.7	122.9	149
pks	ANC non-wilt	116.5	**32.1**	**37.9**	**142.8**	99.2	121.9	**121.9**
pks	non-wilt	104	**34**	**14**	**66**	99	141	141
nrps	ANC wilt	100	20.2	17	58.1	10.1	32	43
nrps	ANC non-wilt	102.3	20.2	20.5	67.8	10.6	**40**	40
nrps	non-wilt	109.4	17	19	62	**9**	52	52
terpene	ANC wilt	56.6	8	6	25.5	19.5	29.7	31
terpene	ANC non-wilt	**61.3**	**12.6**	**10**	28.8	**18.2**	**26.3**	26.3
terpene	non-wilt	**78.1**	**18**	13	22	20	**14**	**14**
other	ANC wilt	90	25.7	12	101	34.9	63.8	51
other	ANC non-wilt	84.8	27.2	**19**	**80.6**	**39.4**	62.9	**62.9**
other	non-wilt	84.3	30	20	96	41	60	60
PL	ANC wilt	33.1	6	7	38	3.7	6.1	8
PL	ANC non-wilt	29.6	6.1	6.2	**29.7**	**4.2**	7.1	7.1
PL	non-wilt	26.8	6	4	38	4	8	8
AA	ANC wilt	129.3	33.2	36	96	46.6	54.8	61
AA	ANC non-wilt	115.6	35.6	37.4	97.8	50.2	63.8	63.8
AA	non-wilt	112.5	34	33	109	**61**	78	78
CBM	ANC wilt	123.5	43.9	39	123.2	48.9	60.3	81
CBM	ANC non-wilt	118.8	43.6	40.9	115.6	51.9	69.1	69.1
CBM	non-wilt	117.5	42	36	139	**60**	69	69
CE	ANC wilt	98.4	17.3	19	57.5	27.8	25.5	27
CE	ANC non-wilt	84.4	19	20.5	53.3	28.2	**32.6**	**32.6**
CE	non-wilt	79.5	19	21	67	33	45	45
V	ANC wilt	76.3	24.3	24	44.3	31.7	34.7	42
V	ANC non-wilt	**63.1**	24.7	24.4	45.8	32.9	38.9	38.9
V	non-wilt	**51.6**	24	22	59	35	44	44
L	ANC wilt	237.8	167.1	165	184.7	172.5	177.3	178
L	ANC non-wilt	251.3	169.8	166.2	184.3	173.2	179.4	179.4
L	non-wilt	**291.2**	173	163	191	174	182	182
N	ANC wilt	3	3	2	3	3	3.6	3
N	ANC non-wilt	3	3	2.3	2.8	3	**3.3**	3.3
N	non-wilt	3	3	2	3	3	3	3
CYP	ANC wilt	151.8	14.1	17	78.7	45.8	54.4	71
CYP	ANC non-wilt	131.6	16.6	19.3	90.3	45.1	62.7	62.7
CYP	non-wilt	118.1	14	15	84	44	72	72

Node; count: 100, 200, 300, 400

Figure S1. Gene counts for the most extreme expansions and contractions of gene classes in seven wilt groups. ANC wilt is the gene count inferred for the most recent common ancestor of wilt species within each group, ANC non-wilt for their most recent non-wilt ancestor, and non-wilt is the gene count of the most closely related non-wilt species or the ancestor of those. Bold values indicate significant (|Z-score| > 2) difference compared to the ANC wilt node. V - defense mechanisms; L - replication, recombination, and repair; N - cell motility; PL - polysaccharide lyases; CE - carbohydrate esterases; CBM - carbohydrate-binding modules; AA - auxiliary activities; pks - polyketide synthases; nrps – non-ribosomal peptide synthetases; SMC – secondary metabolite clusters; CYP – cytochrome p450 oxidases; V, L and N are KOG functions; PKS, NRPS, terpene, other are SMC families; PL, CE, CBM and AA are a CAZy (carbohydrate-active enzyme) families.

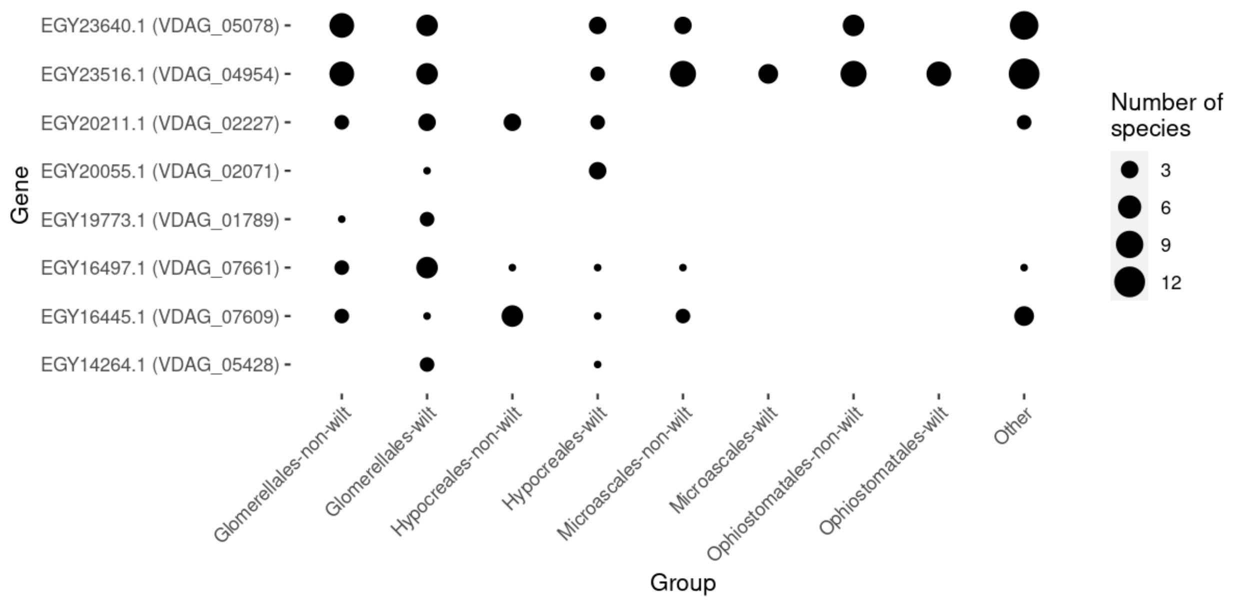

Figure S2. Presence/absence of orthologs of 10 *Verticillium dahliae* genes identified by [27] as unique to *Fusarium* and *Verticillium* wilt species.
